# Effects of high‐intensity exercise training on physical fitness, quality of life and treatment outcomes after oesophagectomy for cancer of the gastro‐oesophageal junction: PRESET pilot study

**DOI:** 10.1002/bjs5.50337

**Published:** 2020-08-28

**Authors:** C. Simonsen, S. Thorsen‐Streit, A. Sundberg, S. S. Djurhuus, C. E. Mortensen, C. Qvortrup, B. K. Pedersen, L. B. Svendsen, P. de Heer, J. F. Christensen

**Affiliations:** ^1^ Centre for Physical Activity Research Rigshospitalet, University of Copenhagen Copenhagen Denmark; ^2^ Departments of Oncology Copenhagen Denmark; ^3^ Surgical Gastroenterology Copenhagen University Hospital Copenhagen Denmark

## Abstract

**Background:**

Treatment for cancer of the gastro‐oesophageal junction (GOJ) can result in considerable and persistent impairment of physical fitness and health‐related quality of life (HRQoL). This controlled follow‐up study investigated the feasibility and safety of postoperative exercise training.

**Methods:**

Patients with stage I–III GOJ cancer were allocated to 12 weeks of postoperative concurrent aerobic and resistance training (exercise group) or usual care (control group). Changes in cardiorespiratory fitness, muscle strength and HRQoL were evaluated. Adherence to adjuvant chemotherapy, hospitalizations and 1‐year overall survival were recorded to assess safety.

**Results:**

Some 49 patients were studied. The exercise group attended a mean of 69 per cent of all prescribed sessions. After exercise, muscle strength and cardiorespiratory fitness were increased and returned to pretreatment levels. At 1‐year follow‐up, the exercise group had improved HRQoL (+13·5 points, 95 per cent c.i. 2·2 to 24·9), with no change in the control group (+3·7 points, −5·9 to 13·4), but there was no difference between the groups at this time point (+9·8 points, −5·1 to 24·8). Exercise was safe, with no differences in patients receiving adjuvant chemotherapy (14 of 16 *versus* 16 of 19; relative risk (RR) 1·04, 95 per cent c.i. 0·74 to 1·44), relative dose intensity of adjuvant chemotherapy (mean 57 *versus* 63 per cent; *P* = 0·479), hospitalization (7 of 19 *versus* 6 of 23; RR 1·41, 0·57 to 3·49) or 1‐year overall survival (80 *versus* 79 per cent; *P* = 0·839) for exercise and usual care respectively.

**Conclusion:**

Exercise in the postoperative period is safe and may have the potential to improve physical fitness in patients with GOJ cancer. No differences in prognostic endpoints or HRQoL were observed. Registration number: NCT02722785 ( https://www.clinicaltrials.gov).

## Graphical abstract

With concurrent aerobic exercise and resistance training twice a week during treatment, patients with cancer of the gastro‐oesophageal junction can return cardiopulmonary fitness and muscle strength to pretreatment levels at completion of treatment. This can be done without affecting prognostic endpoints and with positive effects on health‐related quality of life.

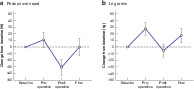

Generally well tolerated and safe.

## Abstract (es)

**Antecedentes:**

El tratamiento del cáncer de la unión gastroesofágica (*gastroesophageal junction*, GEJ) puede determinar un deterioro considerable y persistente de la condición física y de la calidad relacionada con la salud (*health‐related quality of life*, HRQoL). El objetivo de este estudio controlado de seguimiento fue investigar la factibilidad y seguridad del entrenamiento físico postoperatorio.

**Métodos:**

Pacientes con cáncer de GEJ en estadio I‐III fueron asignados a 12 semanas de entrenamiento postoperatorio simultáneo aeróbico y de resistencia o a cuidados médicos habituales. Se evaluaron los cambios en el estado cardiorrespiratoria, fuerza muscular y HRQoL. Se recogieron datos de la adherencia a la quimioterapia adyuvante, hospitalizaciones y supervivencia global a 1 año para evaluar la seguridad.

**Resultados:**

Se estudiaron un total de 49 pacientes. El grupo con ejercicio asistió al 69% de todas las sesiones planificadas. Después del ejercicio, la fuerza muscular y el estado cardiorrespiratorio aumentaron y volvieron a los niveles previos al tratamiento. Si bien al año de seguimiento, el grupo con ejercicio presentó una mejoría de la HRQoL (+13,5 puntos (i.c. del 95% 2,2 a 24,9)), sin cambios en el grupo con atención médica habitual (+3,7 puntos (i.c. del 95% −5,9 a 13,4)), no hubo diferencias entre los grupos en ese momento (+9,8 puntos (i.c. del 95% −5,1 a 24,8)). El ejercicio fue seguro, sin diferencias entre el ejercicio o la atención médica habitual en pacientes que recibían quimioterapia adyuvante 87,5% *versus* 84,2% (RR 1,04 (i.c. del 95% 0,74 a 1,44)), intensidad relativa de la dosis de quimioterapia adyuvante 56,8% *versus* 63,3% (*P* = 0,479), hospitalizaciones 36,8% *versus* 26,1% (RR 1,41 (i.c. del 95% 0,57 a 3,49)) o supervivencia global a 1 año 80,0% *versus* 79,3% (*P* = 0,839).

**Conclusión:**

El ejercicio en el periodo postoperatorio es seguro y puede tener potencial para mejorar la condición física en pacientes con cáncer de GEJ. No se observaron diferencias en los resultados pronósticos o en la HRQoL.

## Introduction

Adenocarcinoma of the gastro‐oesophageal junction (GOJ) is associated with a poor prognosis, often in combination with survivorship issues that include diminished physical function, cardiorespiratory fitness and health‐related quality of life (HRQoL)^1^. Multidisciplinary treatment of GOJ cancer consists of perioperative chemotherapy or chemoradiotherapy, and surgery^2^. During neoadjuvant treatment, patients can experience a marked loss of muscle mass along with worsening cardiorespiratory fitness and HRQoL^3^, ^4^, ^5^, ^6^, ^7^. This loss of physical fitness and HRQoL can be exacerbated subsequently by oesophagectomy, adjuvant chemotherapy and malnutrition^8^, ^9^, ^10^, ^11^. Studies^5^, ^12^, ^13^ have demonstrated that the loss of cardiorespiratory fitness persists for up to 2 years after treatment, muscle mass may continue to decline for up to 1 year after treatment, and HRQoL can be impaired for several years. Low muscle mass and decreased physical function and cardiorespiratory fitness may all lower tolerance to treatment and are associated with a worse prognosis^14^, ^15^. A strong rationale exists for designing interventions aimed at preserving HRQoL and physical fitness in patients with GOJ cancer during treatment.

Structured exercise has been used in other cancer settings, demonstrating improved or maintained HRQoL, muscle function and cardiorespiratory fitness^16^, ^17^. In patients with gastro‐oesophageal cancer, exercise training has been confined primarily to the preoperative setting and after completion of treatment^18^, ^19^, ^20^. In the postoperative setting, patients are recovering from major surgery and many will be undergoing adjuvant chemotherapy. Only two studies^21^, ^22^ have investigated the effect of exercise training in the postoperative period, concurrently with adjuvant treatment in one of these. Both studies used low to moderate intensity, home‐based, unsupervised interventions. There is an absence of evidence regarding the effect of structured and supervised high‐intensity exercise interventions.

The present investigation was a controlled feasibility study to evaluate safety and feasibility of structured preoperative and postoperative concurrent aerobic and resistance training for patients with GOJ cancer. Results from the preoperative intervention have been reported previously^23^. Safety of exercise was assessed as receiving the planned adjuvant treatment, modifications of the adjuvant treatment, hospitalizations and 1‐year overall as well as progression‐free survival. Exercise was considered safe if it did not have a negative effect on adherence to adjuvant treatment or prognosis. Efficacy of exercise was assessed on HRQoL, muscle mass and function, as well as cardiorespiratory fitness. The hypothesis was that exercise would be safe and able to improve physical fitness and HRQoL.

## Methods

The PeRioperativE Study of Exercise Training (PRESET) was a controlled abode‐based feasibility study of exercise in the preoperative and postoperative setting for patients with cancer of the GOJ. The design and primary results, relating to safety and feasibility in the preoperative exercise intervention phase, have been published previously^23^. No power calculation was performed. Instead, the study aimed to include at least 20 participants in each group.

The participants were recruited from the Department of Surgical Gastroenterology at University Hospital Copenhagen between April 2016 and May 2017. Patients with histologically verified GOJ adenocarcinoma, scheduled for intended curative treatment consisting of neoadjuvant chemotherapy or chemoradiotherapy followed by surgery, were eligible for inclusion. Major exclusion criteria were: non‐candidate for intended curative treatment including neoadjuvant therapy, pregnancy, age less than 18 or more than 80 years; any other malignancy requiring active treatment; Eastern Co‐operative Oncology Group performance status above 1; physical disabilities precluding testing or exercise; and inability to read and understand Danish.

Before initiation, the study was approved by the local ethics committee (H‐15014904) and registered at ClinicalTrials.gov (NCT02722785). All participants provided informed written consent before any study‐related procedures were undertaken.

### Group allocation

Participants were allocated without randomization to exercise or usual care during the entire treatment course, based on their address. Participants living within a predefined area of Greater Copenhagen were allocated to the exercise group, and those living in the remaining part of Zealand were allocated to usual care (control group). Residents of Greater Copenhagen were chosen for the exercise group for logistical reasons, as the available exercise facilities were hospital‐based and situated in that area.

#### Usual care

Usual care was provided by clinical dietitians and nurse specialists at Rigshospitalet, Copenhagen, and included information regarding smoking cessation, diet and alcohol, and physical activity guidelines. Participants in the control group were allowed to exercise on their own and to take part in other exercise programmes.

#### Exercise group

In addition to usual care, participants in the exercise group were prescribed 12 weeks of twice‐weekly supervised high‐intensity aerobic and resistance training^23^. If possible, the exercise intervention was initiated 6 weeks after surgery, or otherwise as soon as possible thereafter.

Beginning with aerobic exercise, participants performed a 10‐min warm‐up at 60–70 per cent of maximum heart rate (HR_max_), followed by high‐intensity intervals lasting for 4 min. In sessions 1–4, participants performed three intervals, aiming to reach at least 75 per cent of HR_max_ by the end of each interval. From session 5, participants performed four intervals, aiming to reach 85–95 per cent of HR_max_ after each interval. Between each interval, 3 min of active pause were provided, aiming at a heart rate below 70 per cent of HR_max_. The aerobic exercise was performed on a stationary bicycle or by incline walking on a treadmill. Resistance training consisted of machine‐based leg press, leg extension, seated row and chest press. After a warm‐up set with low intensity, three sets with an increased intensity based on a one‐repetition maximum test (1RM) were performed. In sessions 1–4 the load was set to 50–60 per cent of 1RM and 12 repetitions were performed. In sessions 5–12 the load was increased to 60–70 per cent of 1RM for ten repetitions, and in sessions 13–24 the load was increased to 70–80 per cent of 1RM for eight repetitions. Between each set, 60–90 s of pause were provided. When the target number of repetitions and sets could be performed with more than an estimated one or two repetitions in reserve on the last set, the load was increased in the next session.

An experienced exercise physiologist supervised the exercise sessions and was responsible for adjusting the exercise intervention. Before the start of an exercise session, participants reported fatigue, nausea, pain and dizziness on a yes/no scale. After each session, participants reported whether these symptoms were unchanged, better or worse.

### Standard treatment

All participants received oncological treatment at Rigshospitalet. Most patients received perioperative chemotherapy (Medical Research Council Adjuvant Gastric Infusional Chemotherapy (MAGIC) regimen), consisting of three preoperative cycles of chemotherapy followed by surgery and three postoperative cycles of chemotherapy, each lasting 3 weeks. On day 1 of each cycle, epirubicin (Pharmachemie, Haarlem, the Netherlands) 50 mg/m^2^ was administered intravenously together with either cisplatin (Hospira UK, Hurley, UK) 60 mg/m^2^ (ECX) or oxaliplatin (Fresenius Kabi Oncology, Bordon, UK) 130 mg/m^2^ (EOX). Capecitabine (Accord Healthcare, Harrow, UK) 500 mg/m^2^ was given orally twice daily for 21 days^2^. A smaller proportion of the patients received preoperative chemoradiotherapy (CROSS regimen) with carboplatin (Fresenius Kabi Oncology) (doses were titrated to achieve an area under the curve of 2 mg per ml per min) and paclitaxel (Fresenius Kabi Oncology) 50 mg/m^2^ administered weekly, concurrent with radiotherapy (41·4 Gy in 23 fractions, 5 days per week)^24^.

After the completion of neoadjuvant treatment, participants underwent surgery using a two‐stage Ivor Lewis procedure, performed as robot‐assisted minimally invasive oesophagectomy, hybrid (robot‐assisted laparoscopy combined with thoracotomy) or open (laparotomy and thoracotomy) at the preference of the operating surgeon.

### Assessment of study endpoints

#### Cardiorespiratory fitness, muscle strength and body composition

Participants in the exercise group underwent cardiorespiratory fitness, maximum muscle strength and body composition testing before and after completion of the exercise intervention. Cardiorespiratory fitness was assessed as peak power output (PPO) in an incremental exercise test performed on an electronically braked bicycle ergometer (Monark E839E; Monark, Varberg, Sweden). After a 3‐min warm‐up at 50 W, the intensity was increased by 20 W every minute until exhaustion. PPO was calculated as PPO = Watt_completed_ + 20*(*t*/60), where Watt_completed_ was the last workload fully completed and *t* was the time in seconds completed on the last workload. The HR_max_ achieved during the incremental test was used to prescribe the aerobic exercise.

Maximum muscle strength was measured as 1RM in leg press, leg extension, chest press and seated row. The warm‐up consisted of a series of sets of ten, six, three and one repetition(s), with increasing weight after each set. After each successful attempt the weight was increased, and a 1‐min pause was provided. The highest weight lifted with the correct technique was used as 1RM.

Body composition was assessed using a dual‐energy X‐ray absorptiometry scanner (Lunar Prodigy; Lunar Corporation, Madison, Wisconsin, USA). The whole‐body scan measured total lean mass, appendicular lean mass, fat mass and fat percentage.

#### Health‐related quality of life

HRQoL was assessed using the Functional Assessment of Cancer Therapy – Esophageal (FACT‐E) questionnaire with all its subdomains^25^. Baseline assessment of HRQoL was done at inclusion, after the participants' first visit to the outpatient clinic. Subsequent assessments of HRQoL were scheduled to follow participants' standard treatment‐related follow‐ups at the outpatient clinic. Assessments were scheduled for 1–3 days before surgery, postoperative assessment (approximately 14 days after surgery), 2–6 months and 7–14 months after surgery.

#### Bodyweight and treatment‐related outcomes

During scheduled visits to the outpatient clinic, bodyweight of the participants was measured. Data relating to adjuvant treatment and survival were extracted from the participants' electronic medical records by a blinded oncologist and surgeon respectively. Outcomes relating to adjuvant treatment included the number of participants receiving planned adjuvant chemotherapy, time from surgery to initiation of adjuvant chemotherapy (defined as the number of days from surgery to initiation of adjuvant treatment), and relative dose intensity of the adjuvant chemotherapy^26^. Data on adjuvant treatment were relevant only for the participants scheduled to have adjuvant chemotherapy (the MAGIC regimen) and those who had undergone tumour resection. Hospitalizations were assessed during chemotherapy for the patients receiving adjuvant chemotherapy. For patients not receiving adjuvant chemotherapy, hospitalizations were assessed during a 12‐week period (equivalent to the length of the exercise intervention), beginning 6 weeks after surgery or after discharge if the length of stay after surgery extended beyond 6 weeks. The 1‐year overall survival rate was assessed from inclusion until death from any cause or censoring at 1‐year follow‐up. The 1‐year progression‐free survival rate was assessed from inclusion until first radiologically or histologically verified progression or death from any cause, whichever came first, or censoring at 1‐year follow‐up.

### Statistical analysis

Baseline characteristics, as well as safety of the exercise intervention, were analysed using the unpaired Student's *t* test for continuous variables and the χ^2^ or Fisher's exact test for categorical and ordinal data. Overall and progression‐free survival data were analysed using the log rank test for time‐to‐event comparisons. The effect of the exercise intervention on changes in body composition, muscle strength and cardiorespiratory fitness over time were analysed using a linear mixed model with time as a fixed effect and subject as a random effect adjusted for baseline variables. For changes in bodyweight and HRQoL, group was included as a fixed effect and an interaction between group and time was added to the linear mixed model. Tukey adjustments were used for multiple comparisons. Analyses were conducted using R (The R Foundation for Statistical Computing, Vienna, Austria) and the package lme4^27^. The effect of the exercise intervention and changes in HRQoL and bodyweight are presented as estimated means with 95 per cent c.i. from the linear mixed model. A two‐tailed *P* < 0·050 was considered statistically significant.

## Results

**Fig. 1:**
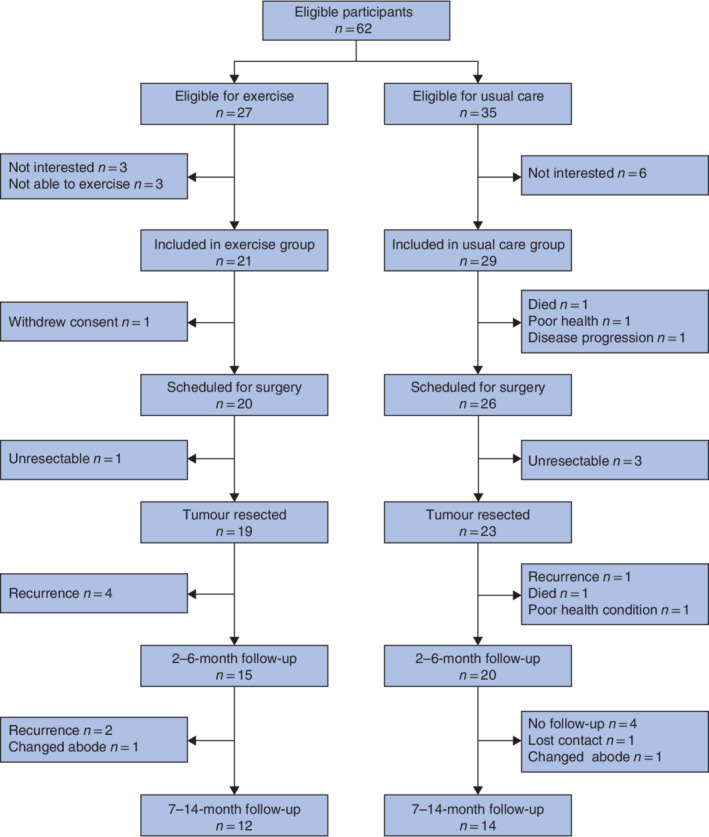
Flow diagram for the study

Of the 50 participants included in the PRESET study, one participant from the exercise group withdrew consent, leaving 49 participants in this follow‐up study (*Fig*. *1*). Baseline characteristics, including disease stage and treatment procedures of the participants remaining in the study, are presented in *Table* *1*. Although not statistically significant, some baseline characteristics were unbalanced between groups at baseline.

**Table 1:** Baseline characteristics

	All patients (*n* = 49)	Exercise group (*n* = 20)	Usual care group (*n* = 29)	*P* §
**Age (years)** *	65·0(7·7)	63·7(8·1)	66·0(7·4)	0·316¶
**Sex ratio (M : F)**	44 : 5	17 : 3	27 : 2	0·387
**BMI (kg/m** ^**2**^ **)** *	28·1(5·5)	28·7(5·5)	27·8(5·6)	0·620¶
**Diabetes**				0·105
Yes	7	5	2	
No	42	15	27	
**Smoker**				0·055
Current	10	1	9	
Previous	28	15	13	
Never	11	4	7	
**Alcohol intake (units/week)**				0·433
≤ 7 or ≤ 14	41	18	23	
> 7 or > 14	8	2	6	
**Physical activity (min MVPA/week)**				0·482
< 150	35	16	19	
≥ 150	14	4	10	
**cTNM stage**				0·522
I	5	2	3	
II	30	14	16	
III	14	4	10	
**Treatment regimen** **†**				0·778
MAGIC EOX	20	8	12	
MAGIC ECX	21	10	11	
CROSS	6	2	4	
**Surgical procedure** **‡**				0·363
RAMIO	2	0	2	
Hybrid	7	2	5	
Open	33	17	16	

* Values are mean(s.d.).

† Data missing data for two subjects owing to death and progression before initiation of treatment.

‡ Seven participants are not included as no surgery performed or tumour not resected. MVPA, moderate to vigorous physical activity; MAGIC, Medical Research Council Adjuvant Gastric Infusional Chemotherapy; EOX, epirubicin plus oxaliplatin; ECX, epirubicin plus cisplatin; RAMIO, robot‐assisted minimally invasive oesophagectomy.

§ χ^2^ or Fisher's exact test, except

¶ unpaired Student's *t* test.

### Adherence and effect of postoperative exercise training

Of 19 participants, 16 initiated the postoperative exercise intervention. One participant did not want to undergo exercise training, contact was lost for one, and the other did not want to engage in postoperative exercise training. In total, 13 participants completed the postoperative exercise training and three discontinued owing to recurrence. All data relating to the feasibility of the exercise intervention are presented in *Table* *S1* (supporting information). The exercise intervention was initiated a mean of 58 (range 44–124) days after surgery. Overall, the participants attended a mean of 16·6 of 24 prescribed sessions (69 per cent), and the 13 participants completing the exercise intervention attended a mean of 19·5 of 24 sessions (81 per cent). In total, 90·4 per cent of all completed aerobic exercise intervals with heart rate data available and 76·6 per cent of all resistance training exercises were completed with planned or higher intensity.

Early termination of a session was necessary once for three participants. Modifications of the planned exercise were necessary at least once for 12 of 16 participants, and 22·6 per cent of all 265 sessions required modification. The most common reasons for modification were musculoskeletal pain and nausea for aerobic exercise, and musculoskeletal pain for resistance training. Self‐reported symptoms were common before exercise: fatigue (38·1 per cent of sessions), nausea (22·3 per cent of sessions), pain (24·9 per cent of sessions) and dizziness (4·2 per cent). The self‐reported symptoms were most frequently unchanged (86·5–96·4 per cent of all sessions) or improved (1·6–11·6 per cent of all sessions) after exercise, but worsened in some subjects (0·8–2·0 per cent of all sessions) (*Table* *S1*, supporting information).

Physiological endpoint results are shown in *Table* *S2* (supporting information). Compared with pretraining values, PPO in the watt‐max test (+30·7 (95 per cent c.i. 16·3 to 45·1) W), leg press 1RM (+23·0 (12·4 to 33·5) kg), leg extension 1RM (+10·4 (5·7 to 15·1) kg), chest press 1RM (+4·8 (2·6 to 6·9) kg) and seated row 1RM (+8·5 (4·2 to 12·8) kg) was increased after exercise training.

**Fig. 2:**
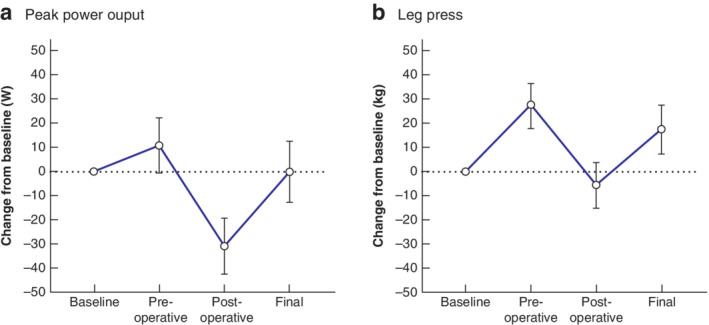
Changes in physical fitness from baseline before and after preoperative and postoperative interventions **a** Estimated mean changes in peak power output from the watt‐max exercise test. **b** Estimated mean changes in muscle strength assessed by one‐repetition maximum test (1RM) leg press. Error bars denote 95 per cent confidence intervals.

After postoperative exercise training, all measures of muscle strength and cardiorespiratory fitness were returned to or improved compared with baseline levels at diagnosis (*Fig*. *2*).

### Bodyweight and body composition

The median duration of follow‐up from surgery to the 2–6‐month assessment was 116 (i.q.r. 105·5 to 141·0) days for the exercise group and 138 (103·0 to 153·0) days for the control group (*P* = 0·223). Median times from surgery to the 7–14‐month follow‐up were 280 (266·8 to 321·0) and 294 (274·8 to 355·3) days for exercise and usual care respectively (*P* = 0·368). From baseline to last follow‐up after 7–14 months, bodyweight decreased by a mean of 9·9 (95 per cent c.i. 6·8 to 13·0) kg in the control group and by 11·3 (7·9 to 14·6) kg in the exercise group (*Table* *2*).

**Table 2:** Safety and tolerability

	Exercise group (*n* = 20)	Usual care group (*n* = 29)	*P* **
**Hospitalization** **§**	*n* = 19	*n* = 23	
No. hospitalized	7	6	1·41 (0·57, 3·49)††
General condition	3	1	
Infection	2	1	
Febrile neutropenia	1	2	
Health concerns	1	0	
Cardiovascular problem	0	2	
**Adjuvant treatment** **¶**	*n* = 16	*n* = 19	
Received adjuvant therapy	14	16	1·04 (0·74, 1·44)††
Time to adjuvant therapy (days)*	56·0 (51·0–57·8)	58·5 (56·8–63·3)	0·628
Relative dose intensity (%)†	57(24)	63(24)	0·479
**Mean weight change (kg)** **‡** **#**			
After surgery	−3·5 (–6·3, −0·6)	−3·2 (−5·9, −0·5)	−0·2 (−4·2, 3·7)‡‡
At 2–6 months	−10·6 (−13·7, −7·4)	−9·9 (−12·7, −7·1)	−0·6 (−4·8, 3·5)‡‡
At 7–14 months	−11·3 (−14·6, −7·9)	−9·9 (−13·0, −6·8)	−1·4 (−6·0, 3·2)‡‡

Values are

* median (i.q.r.) and

† mean(s.d.);

‡ values in parentheses are 95 per cent confidence intervals.

§ Hospitalizations were assessed during adjuvant chemotherapy for patients receiving adjuvant treatment, or during a 12‐week period beginning 6 weeks after surgery or after discharge if hospital stay extended beyond 6 weeks after surgery.

¶ Included only participants treated with the MAGIC regimen who had their cancer resected.

# Included only participants available and assessed during follow‐up (after surgery: 19 in exercise and 21 in control group; at 6–12 months: 15 in exercise and 19 in control group; at 7–14 months: 12 in exercise and 14 in control group).

** *P* value (unpaired Student's *t* test) unless indicated otherwise;

†† relative risk and

‡‡ between‐group difference.

Data on body composition are available in *Table* *S2* (supporting information). During postoperative exercise training participants in the exercise group lost a mean of 3·4 (95 per cent c.i. 1·4 to 5·3) kg fat. No changes were observed in total or appendicular lean mass during postoperative exercise training. Compared with baseline, a mean loss of 3·4 (1·7 to 5·1) kg lean mass, 0·7 (0·4 to 0·9) kg/m^2^ appendicular lean mass, and 7·5 (5·7 to 9·2) kg fat was observed after the postoperative exercise intervention.

### Health‐related quality of life

**Fig. 3:**
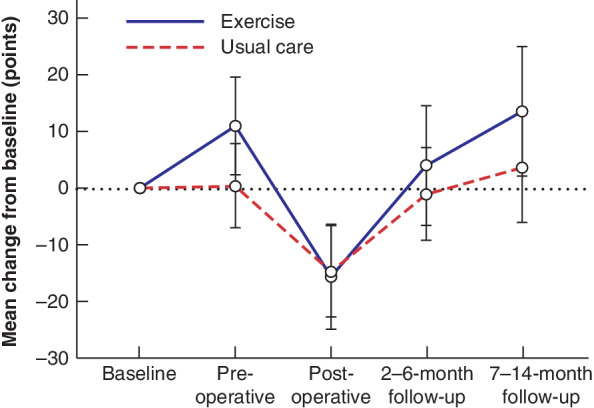
Overview of health‐related quality of life in exercise and usual care groups, assessed as total FACT‐E score from diagnosis to final follow‐up after surgery Error bars denote 95 per cent confidence intervals.

Data on HRQoL assessed by the FACT‐E questionnaire with all its subdomains are available in *Tables* *S3* and *S4* (supporting information). At postoperative follow‐up, both the exercise group (−15·6 (95 per cent c.i. −24·8 to −6·3) points) and the usual care group (−14·6 (−22·6 to −6·5) points) had reductions in the total FACT‐E score compared with baseline, with no difference between the groups (−1·0 (−13·3 to 11·3) points). At 2–6 months' follow‐up, the total FACT‐E score was no different from baseline in either group. The exercise group showed improvement in the total FACT‐E score at the 7–14‐month follow‐up (+13·5 (2·2 to 24·9) points), whereas no changes were observed in the usual care group (+3·7 (−5·9 to 13·4) points) (*Fig*. *3*). There was no between‐group difference for total FACT‐E score at the 7–14‐month follow‐up (+9·8 (−5·1 to 24·8) points).

### Treatment‐related outcomes

**Fig. 4:**
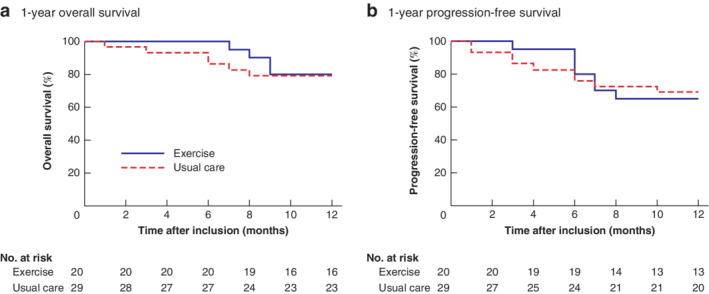
Kaplan–Meier curves of 1‐year overall and progression‐free survival in exercise and usual care groups **a** Overall and **b** progression‐free survival. **a** *P* = 0·839, **b** *P* = 0·875 (log rank test).

Data on safety and tolerability are presented in *Table* *2*. In the exercise group, 14 of 16 participants received planned adjuvant chemotherapy, compared with 16 of 19 in the usual care group (relative risk (RR) 1·04, 95 per cent c.i. 0·74 to 1·44). The median time from surgery to initiation of adjuvant chemotherapy was 56·0 *versus* 58·5 days, and the mean relative dose intensity was 57 *versus* 63 per cent, for exercise and usual care respectively. In the exercise group, seven of 19 participants were hospitalized, compared with six of 23 of those in the usual care group (RR 1·41, 0·57 to 3·49). Overall and progression‐free survival were assessed from baseline to 1‐year follow‐up for all 49 participants. There was no difference in the 1‐year overall survival rate for exercise compared with usual care (16 of 20 (80 per cent) *versus* 23 of 29 (79 per cent) respectively; *P* = 0·839) or in the 1‐year progression‐free survival rate (13 of 20 (65 per cent) *versus* 20 of 29 (69 per cent); *P* = 0·875) (*Fig*. *4*).

## Discussion

This study has shown that high‐intensity exercise can be initiated after surgery concurrent with adjuvant treatment, and that cardiorespiratory fitness and muscle strength returned to pretreatment levels. HRQoL based on the total FACT‐E score and other subdomains at 7–14 months' follow‐up increased only in the exercise group, although the changes were no different from those in the usual care group. These findings are of clinical importance as several studies^8^, ^12^, ^13^, ^28^, ^29^ have reported that physical fitness and HRQoL are impaired in the long term after cessation of treatment, and do not recover.

In line with the findings of the present study, previous studies^20^, ^22^, ^30^ have demonstrated that exercise in the postoperative period and after completion of treatment can improve cardiorespiratory fitness in patients with gastro‐oesophageal cancer, although the role of high‐intensity exercise has not been explored previously during adjuvant chemotherapy. An important finding was the relatively good adherence to exercise training during adjuvant chemotherapy, which is often poorly tolerated compared with neoadjuvant treatment^2^. The participants generally exercised with the prescribed intensity in both aerobic exercise and resistance training, although one in five sessions needed modification to allow completion and three sessions had to be terminated early. These findings imply that, although high‐intensity exercise in the postoperative period is safe and feasible, it should be supervised by experienced instructors to ensure that required adjustments can be made to accommodate changes in well‐being and performance from day to day or during exercise.

Weight loss after oesophagectomy is common and can be caused by malnutrition, dysphagia and the surgical resection^10^, ^31^. As a consequence, patients with GOJ cancer often experience a substantial loss of muscle mass, associated with impaired overall survival^15^. An important consideration, therefore, relating to postoperative exercise for patients with gastro‐oesophageal cancer, is potentially exacerbating weight loss as the increased energy expenditure during exercise may add to an already existing caloric deficit. Exacerbated weight loss in the exercise group was not seen, however, compared with usual care at any follow‐up assessment in the present study. Notably, during the 12 weeks of postoperative exercise, patients in the exercise group lost an estimated 3·4 kg fat, but neither total (+1·0 kg) nor appendicular (+0·1 kg/m^2^) lean mass decreased.

Most participants in the present study conducted their exercise training during adjuvant treatment, and it was therefore important to ensure that, in an attempt to mitigate side‐effects of treatment, the training did not interfere negatively with the treatment. Despite its relevance, this is rarely investigated and reported in exercise oncology studies^32^, ^33^. For all treatment outcomes, no differences existed between exercise or usual care, suggesting that exercise did not affect adherence to the oncological treatment. Similarly, there was no difference between exercise or usual care in 1‐year overall or progression‐free survival. Participants in the exercise group had a higher rate of hospitalization (RR 1·41, 95 per cent c.i. 0·57 to 3·49) compared with those in the usual care group during the postoperative exercise intervention period. This was in contrast to the present authors' finding in the preoperative period, where the rate of hospitalization in the exercise group was lower than that in the usual care group^23^. This difference could be explained by imbalance in presurgical dropouts (1 in the exercise group *versus* 6 in the control group); postoperative outcomes including hospitalizations may have been influenced if a larger proportion of potentially high‐risk patients did not undergo surgery in the usual care group.

The present study has several limitations. Allocation to exercise or usual care was based on address rather than randomization, reflecting availability of exercise facilities in the Greater Copenhagen area. Without randomization there may have been systematic differences between the groups. As physical fitness in the usual care group was not assessed, it cannot be ruled out that the return to baseline values was an effect of recovery, although other studies^8^, ^12^, ^28^ have demonstrated that loss of physical fitness in response to treatment for GOJ cancer persists after treatment has ended.

High‐intensity exercise in the postoperative period is safe. Increases in cardiorespiratory fitness, muscle strength and HRQoL were achieved with adherence to concurrent adjuvant treatments in patients with cancer of the gastro‐oesophageal junction.

## Supporting information


**Appendix S1.** Tables.
