# Supplementary material for: Effects of high‐intensity exercise training on physical fitness, quality of life and treatment outcomes after oesophagectomy for cancer of the gastro‐oesophageal junction: PRESET pilot study
Source: BJS Open. 2020 Aug 28;4(5):855–64. doi: 10.1002/bjs5.50337 (PMC7528530; doi:10.1002/bjs5.50337)
Supplement: Supplementary file 1 — Appendix S1. Tables. [file BJS5-4-855-s001.docx]

**BJS5_50337**

**Effects of high-intensity exercise training on physical fitness, quality of life and treatment outcomes after oesophagectomy for cancer of the gastro-oesophageal junction: PRESET pilot study**

**C. Simonsen, S. Thorsen-Streit, A. Sundberg, S. Sigmundsdóttir Djurhuus, C. Ehlers Mortensen, C. Qvortrup, B. Klarlund Pedersen, L. B. Svendsen, P. de Heer and J. F. Christensen**

**Table S1** Feasibility of postoperative exercise

| **Attendance** | Mean (SD) | Attendance rate [Range] |
| --- | --- | --- |
| - All participants, n=16 - None-dropouts, n=13 | 16.6 (7.0)  19.5 (3.0) | 69.0% [8.3%-100%]  81.4% [54.2%-100%] |
| **Permanent discontinuation** | No of patients (%) | Reasons for discontinuation |
| - No | 13 (82.4%) |  |
| - Yes | 3 (18.8%) | Recurrence (100%) |
| **Exercise cancelations** | No of patients (%) | No. sessions (% of possible) |
| Sessions cancelled | 15 (93.8%) | 70 (20.9%) |
| - Health-related | 10 (62.5%) | 36 (10.7%) |
| - Motivation | 4 (25.0%) | 7 (2.1 %) |
| - Injury | 3 (18.8%) | 6 (1.8%) |
| - Hospitalized | 4 (25.0%) | 7 (2.1%) |
| - Treatment | 3 (18.8%) | 5 (1.5%) |
| - Vacation | 7 (43.8%) | 9 (2.7%) |
| Exercise interruption^†^ | No of patients (%) | No. of interruptions |
| - Without | 8 (50%) |  |
| - With ≥1 | 8 (50%) | 11 |
| **Exercise modification*** | No. of patients (%) | No. sessions (% of 265 sessions) |
| With ≥1 modification | 12 (71%) | 60 (22.6%) |
| Dose-reductions | 12 (75.0%) | 67 |
| *Aerobic exercise* | 9 (56.3%) | 40 (15.1%) |
| - Nausea | 2 (12.5%) | 10 (3.8%) |
| - Muscle-skeletal pain | 3 (18.8%) | 11 (4.2%) |
| - Motivational | 1(6.3%) | 6 (2.3%) |
| - Other | 2 (12.5%) | 6 (2.3%) |
|  |  |  |
| *Resistance training* | 8 (50%) | 27 (10.2%) |
| - Muscle-skeletal pain | 6 (37.5%) | 22 (8.3%) |
| - Nausea | 1 (6.3%) | 2 (0.8%) |
| - Fatigue | 2 (12.5%) | 3 (1.1%) |
|  |  |  |
| Early termination | 3 (18.8%) | 3 (1.1%) |
| - Fatigue | 1 (6.3%) | 1 (0.4%) |
| - Muscle-skeletal pain | 1 (6.3%) | 1 (0.4%) |
| - Nausea | 1 (6.3%) | 1 (0.4%) |
| **Self-reported Symptoms^¤^** | No. of patients (%) | No. sessions (% sessions) |
| Fatigue (Pre-training) | 14 (87.5%) | 101 (38.1%) |
| - Worsened |  | 5 (2.0%) |
| - Unchanged |  | 217 (86.5%) |
| - Improved |  | 29 (11.6 %) |
| Nausea (Pre-training) | 10 (62.5%) | 59 (22.3%) |
| - Worsened |  | 4 (1.6%) |
| - Unchanged |  | 224 (88.9%) |
| - Improved |  | 24 (9.5%) |
| Pain (Pre-training) | 12 (75.0%) | 66 (24.9%) |
| - Worsened |  | 2 (0.8%) |
| - Unchanged |  | 237 (94.4%) |
| - Improved |  | 12 (4.8%) |
| Dizziness (Pre-training) | 5 (31.3%) | 11 (4.2%) |
| - Worsened |  | 5 (2.0%) |
| - Unchanged |  | 244 (96.4%) |
| - Better |  | 4 (1.6%) |
| **Aerobic exercise adherence^¶^** | Planned intensity achieved  No. of intervals (%) | Planned intensity not achieved  No. of intervals (%) |
| Session 1 to 4 (Above 75% HR_max_): |  |  |
| - Interval 1 | 52 (96.3%) | 2 (3.7%) |
| - Interval 2 | 53 (100%) | 0 (0%) |
| - Interval 3 | 46 (100%) | 0 (0%) |
| Session 5 to 24 (Above 85% HR_max_): |  |  |
| - Interval 1 | 142 (81.6%) | 32 (18.4%) |
| - Interval 2 | 159 (90.3%) | 17 (9.7%) |
| - Interval 3 | 158 (90.3%) | 17 (9.7%) |
| - Interval 4 | 158 (91.9%) | 14 (8.1%) |
| Total Aerobic Exercise | 768 (90.4%) | 82 (9.7%) |
| **Resistance training adherence^§^** | Planned intensity achieved  No. of sessions (%) | Planned intensity not achieved  No. of sessions (%) |
| Leg press |  |  |
| - Session 1 to 4 (50% 1RM) | 61 (100%) | 0 (0%) |
| - Session 5 to 12 (60% 1RM) | 97 (92.3%) | 8 (7.7%) |
| - Session 13 to 24 (70% 1RM) | 78 (90.7%) | 8 (9.3%) |
| Leg extension |  |  |
| - Session 1 to 4 (50% 1RM) | 35 (77.8%) | 10 (22.2%) |
| - Session 5 to 12 (60% 1RM) | 80 (97.6%) | 2 (2.4%) |
| - Session 13 to 24 (70% 1RM) | 45 (71.4%) | 18 (28.6%) |
| Chest press |  |  |
| - Session 1 to 4 (50% 1RM) | 17 (32.7%) | 35 (67.3%) |
| - Session 5 to 12 (60% 1RM) | 34 (35.4%) | 62 (64.6%) |
| - Session 13 to 24 (70% 1RM) | 30 (37.0%) | 51 (63.0%) |
| Seated Row |  |  |
| - Session 1 to 4 (50% 1RM) | 50 (96.2%) | 2 (3.8%) |
| - Session 5 to 12 (60% 1RM) | 94 (98.9%) | 1 (1.1%) |
| - Session 13 to 24 (70% 1RM) | 71 (83.5%) | 14 (16.5%) |
| Total Resistance Training | 692 (76.6%) | 211 (23.4%) |

†, missing two consecutive sessions; * Exercise modified from prescribed by changing intensity, duration, number of sets, repetitions or exercises; ¤ Symptoms, Assessment of symptoms prior to and after exercise on a yes/no scale before exercise and a worsened/unchanged/improved scale after exercise; **¶** 26 intervals (3% of all intervals) are missing heart rate data due to equipment malfunction (n=11), other participants heart rate was registered by the equipment (n=3) and for unknown reasons (n=12), **§** Numbers differs between exercises due to some participants not performing specific exercises due to modifications or muscle-skeletal pain

**Table S2** Effect of postoperative exercise training

|  | **Preoperative Exercise*** | | **Postoperative Exercise** | | **Pre – post training** | | **Difference from baseline** | |
| --- | --- | --- | --- | --- | --- | --- | --- | --- |
|  | Baseline  (n=20) | Post training  (n=18) | Pre training  (n=17) | Post training  (n=13) | Preoperative* | Postoperative | Postoperative | Final |
| **Cardiorespiratory fitness** | | | | | | | | |
| Watt-max (Watt) | 150.9 (60.5) | 165.2 (52.1) | 126.5 (42.3) | 162.5 (63.6) | 10.8  (-0.6, 22.1) | 30.7  (16.3, 45.1) | -30.8  (-42.3, -19.2) | -0.1  (-12.6, 12.4) |
| **Muscle strength – 1 repetition maximum** | | | | | | | | |
| Leg press (kg) | 117.3 (28.3) | 143.9 (30.3) | 111.8 (23.9) | 137.3 (33.4) | 27.0  (17.7, 36.2) | 23.0  (12.4, 33.5) | -5.7  (-15.2, 3.7) | 17.2  (7.2, 27.3) |
| Leg extension (kg) | 50.5 (11.0) | 60.4 (13.1) | 50.2 (9.7) | 60.5 (11.5) | 9.9  (6.2, 13.7) | 10.4  (5.7, 15.1) | -1.4  (-5.2, 2.5) | 9.0  (5.0, 13.0) |
| Chest press (kg) | 31.6 (9.9) | 36.8 (12.8) | 28.7 (10.5) | 35.0 (12.8) | 5.2  (3.3, 7.1) | 4.8  (2.6, 6.9) | -3.1  (-5.1, -1.1) | 1.7  (-0.4, 3.8) |
| Seated row (kg) | 59.6 (14.5) | 68.9 (16.5) | 54.1 (13.8) | 63.7 (17.8) | 8.9  (5.1, 12.6) | 8.5  (4.2, 12.8) | -4.9  (-8.8, -1.0) | 3.7  (-0.4, 7.7) |
| **Body composition** | | | | | | | | |
| Lean body mass (kg) | 56.0 (10.5) | 57.0 (10.7) | 52.9 (8.9) | 54.5 (6.3) | -0.2  (-1.6, 1.3) | 1.0  (-1.6, 3.6) | -4.4  (-5.9, -2.8) | -3.4  (-5.1, -1.7) |
| Appendicular lean mass (kg/m^2^) | 7.8 (0.9) | 7.8 (1.01) | 7.1 (0.71) | 7.3 (0.72) | 0.0  (-0.3, 0.2) | 0.1  (-0.3, 0.5) | -0.8  (-1.0, -0.5) | -0.7  (-0.9, -0.4) |
| Fat mass (kg) | 30.3 (13.4) | 32.0 (12.5) | 27.9 (12.0) | 23.4 (10.3) | -0.2  (-1.8, 1.5) | -3.4  (-5.3, -1.4) | -4.1  (-5.8, -2.4) | -7.5  (-9.2, -5.7) |
| Fat percentage (%) | 34.0 (9.4) | 35.1 (7.6) | 33.9 (8.0) | 28.8 (8.1) | -0.3  (-2.0, 1.3) | -3.9  (-5.7, -2.1) | -1.4  (-3.1, 0.3) | -5.3  (-7.1, -3.5) |

Values are raw means (SD) or estimated means mean (95% confidence interval). * Data related to the pre-operative intervention are previously published.

**Table S3** Health-related quality of life FACT-E within-group changes

|  | **Exercise** | | | | | **Usual Care** | | | | |
| --- | --- | --- | --- | --- | --- | --- | --- | --- | --- | --- |
|  | **Mean**  **(SD)** | **Change scores from baseline** | | | | **Mean**  **(SD)** | **Change scores from baseline** | | | |
| **FACT-E** | Baseline  (n = 19) | Pre-operative*  (n = 19) | Post-operative  (n = 16) | 2-6 mos.  Follow-up  (n = 12) | 7-14 mos. follow-up  (n = 10) | Baseline  (n= 29) | Pre-operative*  (n = 25) | Post-operative  (n = 21) | 2-6 mos.  Follow-up  (n = 20) | 7-14 mos. follow-up  (n = 14) |
| Physical well being | 23.8  (3.3) | 0.7  (-1.4, 2.7) | -7.9  (-10.2, -5.7) | -3.7  (-6.2, -1.1) | 0.2  (-2.5, 3.0) | 23.7  (4.3) | -2.0  (-3.8, -0.2) | -6.8  (-8.7, -4.8) | -3.2  (-5.2, -1.2) | -0.7  (-3.0, 1.6) |
| Social well being | 25.1  (3.3) | 0.2  (-1.4, 1.8) | -0.5  (-2.2, 1.2) | -0.8  (-2.7, 1.1) | -0.4  (-2.5, 1.6) | 24.1  (3.6) | -0.3  (-1.7, 1.1) | 0.3 (-1.2, 1.7) | -0.9  (-2.5, 0.6) | -2.6  (-4.4, -0.9) |
| Emotional well being | 16.3  (3.2) | 2.6  (1.1, 4.1) | 3.8  (2.2, 5.4) | 4.1  (2.3, 5.8) | 2.9  (1.0, 4.9) | 17.7  (4.5) | 0.9  (-0.4, 2.2) | 0.8  (-0.5, 2.2) | 2.1  (0.7, 3.5) | 1.0  (-0.6, 2.7) |
| Functional  well being | 20.4  (4.8) | -0.5  (-2.7, 1.7) | -4.5  (-6.9, -2.2) | -1.2  (-3.8, 1.4) | 0.8  (-2.1, 3.6) | 20.1  (5.9) | -0.5  (-2.4, 1.4) | -5.5  (-7.5, -3.5) | -0.8  (-2.9, 1.3) | 0.7  (-1.7, 3.2) |
| Esophagus cancer subscale | 48.4  (10.0) | 7.9  (3.7, 12.1) | -6.7  (-11.2, -2.1) | 4.8  (-0.2, 9.9) | 10.1  (4.4, 15.8) | 50.3  (9.6) | 2.4  (-1.3, 6.0) | -3.5  (-7.4, 0.5) | 2.1  (-2.0, 6.2) | 5.7  (0.8, 10.5) |
| Trial Outcome Index | 92.6  (13.8) | 8.2  (1.2, 15.4) | -18.8  (-26.5, -11.2) | 0.5  (-8.2, 9.3) | 11.2  (1.7, 20.7) | 94.0  (16.5) | -0.2  (-6.4, 6.0) | -15.8  (-22.5, -9.1) | -2.0  (-8.9, 4.8) | 5.4  (-2.6, 13.5) |
| FACT-G | 85.6  (9.5) | 2.9  (-2.5, 8.3) | -9.2  (-15.0, -3.5) | -1.4  (-7.8, 5.1) | 3.4  (-3.6, 10.4) | 85.6  (14.7) | -1.8  (-6.4, 2.9) | -11.1  (-16.0, -6.2) | -2.9  (-8.0, 2.3) | -1.6  (-7.5, 4.4) |
| Total score | 133  (15.2) | 11.1  (2.5, 19.6) | -15.6  (-24.8, -6.3) | 4.0  (-6.5, 14.5) | 13.5  (2.2, 24.9) | 135.8  (20.9) | 0.4  (-7.0, 7.9) | -14.6  (-22.6, -6.5) | -0.9  (-9.1, 7.3) | 3.7  (-5.9, 13.4) |

Values are mean (95% confidence interval) unless stated otherwise. FACT-E/G, Functional Assessment of Cancer Therapy – Esophagus/General. * Data related to the pre-operative intervention are previously published.

**Table S4** Health-related quality of life FACT-E between-group differences

|  | **Between-group changes**  (Exercise - Usual Care) | | | |
| --- | --- | --- | --- | --- |
| **FACT-E** | Pre-operative*  (n = 44) | Post-operative  (n = 37) | 2-6 mos. follow-up  (n = 32) | 7-14 mos. follow-up  (n = 24) |
| Physical well being | 2.7  (-0.1, 5.4) | -1.2  (-4.1, 1.8) | -0.4  (-3.6, 2.8) | 0.9  (-2.7, 4.5) |
| Social well being | 0.5  (-1.6, 2.6) | -0.7  (-3.0, 1.5) | 0.2  (-2.3, 2.6) | 2.2  (-0.5, 4.9) |
| Emotional well being | 1.7  (-0.3, 3.7) | 2.9  (0.8, 5.1) | 2.0  (-0.3, 4.2) | 1.9  (-0.6, 4.4) |
| Functional  well being | -0.1  (-3.0, 2.8) | 0.9  (-2.2, 4.0) | -0.4  (-3.7, 3.0) | 0.0  (-3.8, 3.8) |
| Esophagus cancer subscale | 5.5  (-0.1, 11.1) | -3.2  (-9.2, 2.8) | 2.7  (-3.7, 9.2) | 4.4  (-3.1, 11.9) |
| Trial Outcome Index | 8.4  (-0.1, 17.8) | -3.0  (-13.2, 7.2) | 2.6  (-8.6, 13.7) | 5.8  (-6.7, 18.3) |
| FACT-G | 4.7  (-2.5, 11.8) | 1.9  (-5.7, 9.5) | 1.5  (-6.8, 9.8) | 5.0  (-4.2, 14.1) |
| Total score | 10.6  (-0.7, 22.0) | -1.0  (-13.3, 11.3) | 4.9  (-8.5, 18.2) | 9.8  (-5.1, 24.8) |

Values are mean (95% confidence interval). FACT-E/G, Functional Assessment of Cancer Therapy – Esophagus/General. * Data related to the pre-operative intervention are previously published.
